# Maturity-Onset Diabetes of the Young Type 3 (MODY 3): A Rare Presentation of Diabetes in Primary Care

**DOI:** 10.7759/cureus.63119

**Published:** 2024-06-25

**Authors:** Mariana Mendonça, Paulo Barros, Liliana Santa Cruz, Ana C Pastilha, Rita Cordeiro

**Affiliations:** 1 Family Medicine, Unidade de Saúde Familiar (USF) Mondego, Coimbra, PRT; 2 Family Medicine, Unidade de Saúde Familiar (USF) Mealhada, Mealhada, PRT; 3 Family Medicine, Unidade de Saúde Familiar (USF) Coimbra Sul, Coimbra, PRT

**Keywords:** mody, maturity-onset diabetes of the young, case report, genogram, diabetes, primary healthcare

## Abstract

Maturity-onset diabetes of the young (MODY) is a genetic form of diabetes with an autosomal dominant pattern of transmission characterized by dysfunction in pancreatic β-cells. MODY type 3 (MODY 3) is caused by heterozygous mutations in the hepatocyte nuclear factor 1-α (HNF1A) gene and is sensitive to treatment with sulfonylureas. This case report approaches the diagnostic journey of a 46-year-old woman who was initially misdiagnosed with type 2 diabetes. Despite adherence to pharmacological and lifestyle interventions, her glycemic control deteriorated. A comprehensive family history revealed a strong familial prevalence of diabetes. Genetic testing confirmed MODY 3, leading to the initiation of sulfonylurea therapy and subsequent glycemic control. This case emphasizes the diagnostic hurdles associated with MODY in primary care and the critical role of a genogram analysis in revealing familial patterns and giving strategies for personalized treatment.

## Introduction

Maturity-onset diabetes of the young (MODY) is a genetic form of diabetes with an autosomal dominant pattern of transmission. It is caused by a dysfunction in the pancreatic β-cells and is the most prevalent type of monogenic diabetes. The clinical presentation of MODY may resemble features of both type 1 and type 2 diabetes, making genetic confirmation a critical step in the diagnostic process. Currently, MODY accounts for approximately 1-5% of all diabetes cases [1].

Currently, 14 different subtypes of MODY have been identified by molecular diagnostics [1]. MODY type 3 (MODY 3) is one of those and is caused by heterozygous mutations in the hepatocyte nuclear factor 1-α (HNF1A) gene. Heterozygous carriers of the HNF1A mutation experience a gradual decline in β-cell function, leading to the onset of diabetes in early adulthood [2]. These mutations achieve a high level of penetrance, with approximately 63% of carriers developing diabetes before age 25 [3]. This genetic mutation causes patients to have a profound impairment in insulin secretion but preserves sensitivity to treatment with sulfonylureas. Furthermore, it causes a decrease in the renal threshold for glucose reabsorption. In rare family cases, MODY 3 has also been linked to the occurrence of liver adenomatosis [4].

Due to the potential for hyperglycemia, individuals with MODY 3 are at a similar risk of microvascular and macrovascular complications as patients with type 1 and type 2 diabetes. While MODY 3 patients are not susceptible to ketoacidosis, maintaining tight metabolic control remains important to prevent chronic diabetic complications [2,3].

The first-line treatment recommended for patients with MODY 3 is a lifestyle modification, such as a low-carbohydrate diet, as well as treatment with sulfonylurea, which has remarkable effectiveness in these cases. When properly managed, it allows for adequate and prolonged glycemic control. Other medications used in type 2 diabetes, such as metformin and SGLT-2 inhibitors, are not effective in treating MODY 3. Nevertheless, it's important to be aware that, as the condition progresses, some patients may eventually require insulin treatment [2].

## Case presentation

The patient is a 46-year-old woman with a past history of high blood pressure and was at that time medicated with perindopril 10 mg + amlodipine 5 mg once daily. During both pregnancies, she had gestational diabetes, which reverted fully postpartum. She had no other known medical conditions.

In May 2021, she came to her family physician for a routine follow-up appointment. The patient did not report any classical symptoms of diabetes, such as polydipsia, polyuria, or weight loss. A physical examination showed a blood pressure of 128/75 mmHg and a BMI of 22.3 kg/m2. The patient also brought results of bloodwork previously issued at the last appointment (Table 1). Her bloodwork showed elevated fasting blood glucose, with a value of 142 mg/dl, not present before.

**Table 1:** Results of complete blood count and biochemical analyses in May 2021 HDL-C: high-density lipoprotein-cholesterol; LDL-C: low-density lipoprotein-cholesterol

Analytical parameter	Result	Reference value
Hemoglobin	13.1 g/dL	12.0-16.0 g/dL
Erythrocytes	4.3 x 10^12^/L	4.0-5.2 x 10^12^/L
Platelets	155 x 10^9^/L	150-400 x 10^9^/L
Leukocytes	4.4 x 10^9^/L	3.9-7.7 x 10^9^/L
Glucose	142 mg/dL	70-110 mg/dL
Creatinine	0.82 mg/dL	0.55-1.02 mg/dL
Aspartate aminotransferase	11 U/L	<31U/L
Alanine aminotransferase	15 U/L	<34 U/L
Gamma-glutamyl transferase	8 U/L	<38 U/L
Alkaline phosphatase	67 U/L	30-120 U/L
Triglycerides	87 mg/dL	35-160 mg/dL
Total cholesterol	178 mg/dL	<200mg/dL
LDL-C	102 mg/dL	<160 mg/dL
HDL-C	59 mg/dL	30-70 mg/dL

Subsequent repeat fasting blood glucose, one month later, was also elevated, with a value of 136 mg/dl, along with a glycated hemoglobin (HbA1c) level of 7%, leading to a definitive diagnosis of diabetes.

The patient was then started on pharmacological treatment with metformin 1000 mg once daily, with lifestyle changes such as dietary care and physical exercise also being encouraged. Three months later, she returned for a follow-up appointment with an HbA1c of 7.3%, indicating no improvement. The dose of metformin was doubled to 1000 mg twice daily, and dapagliflozin 10 mg was added. However, HbA1c levels continued to worsen, reaching 8.6%. The patient reported adhering to all prescribed pharmacological therapy, as well as following a Mediterranean low-carbohydrate diet and engaging in moderate-intensity physical exercise four times a week.

Given the worsening control of the patient’s diabetes, a more precise history was taken. When asked if she had ever had abnormal blood glucose values before, she reported that she began experiencing abnormal blood glucose levels at the age of 15, which were successfully managed and normalized solely through lifestyle changes. She also mentioned that during both of her pregnancies, she had gestational diabetes yet maintained excellent glycemic control with no need for pharmacological intervention.

**Figure 1:**
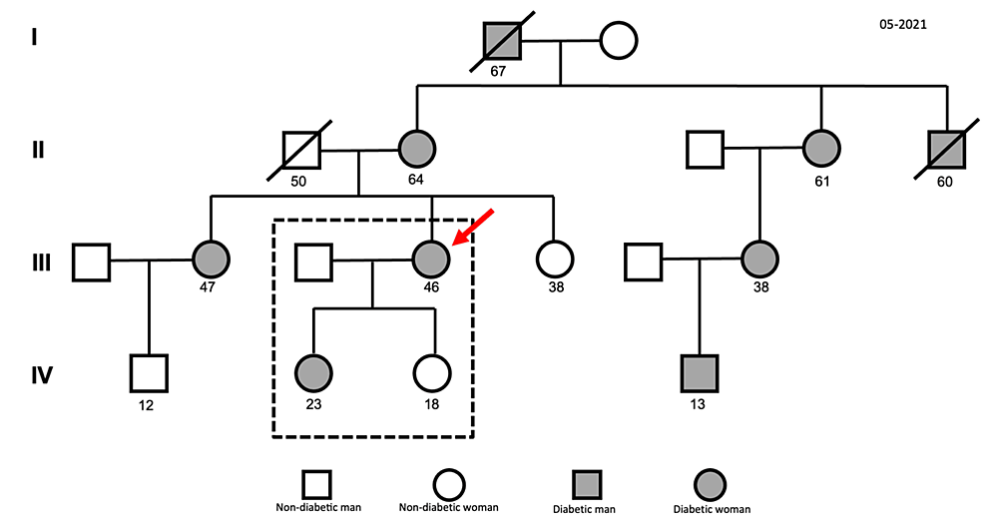
Family genogram initiated in May 2021 with the respective legend The genogram illustrates the presence of diabetes across all generations in the patient's family. Each symbol represents a family member, with those affected by diabetes indicated in gray. The patient is highlighted with a red arrow.

As for her family history, the patient also reported a high prevalence of diabetes in her family. Thus, during the appointment, a family genogram was created (Figure 1). All generations of the family had diabetes. The patient also mentioned that her eldest daughter was diagnosed with diabetes at the age of 11, and a confirmed heterozygous mutation was identified in the HNF1A gene.

Therefore, because of her strong family history of diabetes and failure to lower the HbA1c level with adequate treatment, in October 2022, she was referred to an endocrinologist. At the endocrinology appointment, it was suspected that she might have the same mutation as her daughter, and genetic tests were ordered, which confirmed her diagnosis of MODY 3 (Table 2). Subsequently, she started sulfonylurea (gliclazide 30 mg) once daily and has since achieved and maintained adequate glycemic control. The last HbA1c was 6.6% as of November 2023.

**Table 2:** Genetic analyses in January 2023

Gene	Result
Hepatocyte nuclear factor 1-α - cromossoma 12 (12q24.31)	Heterozygous

It is also important to note that the patient has no history of micro- or macrovascular complications and does not present with any other relevant medical conditions. She continues to undergo annual screening for diabetic retinopathy, nephropathy, and neuropathy.

## Discussion

The diagnosis of MODY poses a challenge for family physicians due to its heterogeneous presentation and low prevalence, often confused with the more prevalent types of diabetes, and, as such, many cases of MODY are misclassified or remain undiagnosed. Specifically, in MODY 3, individuals with the HNF1A mutation may present with normal plasma glucose levels and only glycosuria [1,5]. However, as time goes by, fasting glucose levels tend to increase with age due to progressive loss of beta cell function [3,5]. Furthermore, testing for this disease poses an additional challenge, as the definitive diagnosis is based on expensive molecular genetic tests, often unavailable in a primary care setting [6].

In this clinical case, the presumed diagnosis was type 2 diabetes, due to its high prevalence and the patient's age. For this reason, she was initially treated with metformin and later with dapagliflozin, without any improvement and even with progressively worsening HbA1c levels. The lack of response to therapy combined with the strong family history revealed by the genogram led the family physician to broaden the differential diagnosis beyond the more common type 2 diabetes.

MODY has an autosomal dominant pattern of inheritance [1]. Therefore, the children of a person affected by this type of diabetes have a 50% chance of inheriting the disease. Often, affected family members are unaware they have MODY diabetes; some may be mistakenly diagnosed with type 1 diabetes, especially if diabetes develops before the age of 25, or type 2 diabetes. Whenever there are many family members with diabetes in a family, especially if it is not associated with obesity, the possibility of MODY should be considered [5].

An accurate differential diagnosis is also important due to treatment differences. At first, as with all types of diabetes, a low-carbohydrate diet may be sufficient for glycemic control, especially when HbA1c levels are below 6.5%. However, due to a higher response to sulfonylureas in MODY 3, low doses of these drugs are recommended as first-line pharmacological treatment when glycemic control is inadequate. As hypoglycemia is a common adverse reaction to these drugs, newly diagnosed MODY 3 patients require tighter glucose monitoring. In cases of treatment failure, insulin is another option [1,2,5].

The genogram is an essential tool for the primary care physician. It is a visual representation of a patient’s family history and helps identify hereditary patterns of disease, genetic risk factors, and potential therapeutic interventions. It not only facilitates diagnosis and therapeutic decision-making but also enhances risk communication with both the patient and their family [7].

In this case, the genogram offered important insight into the prevalence and hereditary pattern of diabetes in the patients’ family, hinting at a genetic cause for the patient’s diagnosis, which was confirmed later as MODY 3.

## Conclusions

MODY is an often-overlooked form of diabetes in primary care. Given its similarities but also distinct features in relation to other types of diabetes, it is important that family physicians recognize this disease to allow for timelier referral, diagnosis, and adequate treatment.

One of the tools that can help to recognize this type of hereditary disease is the genogram. By creating a visual representation of a patient’s medical history, it facilitates the detection of patterns of inheritance such as autosomal recessive, dominant, or other clusters of related diseases or risk factors. Therefore, the genogram allows for earlier detection, prognostic assessment, and improved genetic counseling for a patient and their family.
